# Listening to a Consonant Chord Progression during Live Face-to-Face Gaze Enhances Neural Activity in Social Systems

**DOI:** 10.1523/JNEUROSCI.1116-25.2026

**Published:** 2026-03-05

**Authors:** Dash A. Watts, AZA Stephen Allsop, Simone Compton, Xian Zhang, J. Adam Noah, Joy Hirsch

**Affiliations:** ^1^Department of Psychiatry, Howard University, Washington, DC 20060; ^2^Center for Collective Healing, Department of Psychiatry and Behavioral Sciences, Howard University, Washington, DC 20060; ^3^Departments of Neuroscience, Yale University School of Medicine, New Haven, Connecticut 06511; ^4^Comparative Medicine, Yale University School of Medicine, New Haven, Connecticut 06511; ^5^Wu Tsai Institute, Yale University, New Haven, Connecticut 06520

**Keywords:** functional near-infrared spectroscopy (fNIRS), live hyperscanning, music therapy, neuroimaging, social cognition

## Abstract

Although music has been associated with increased prosocial behavior, the underlying mechanisms for music-facilitated social benefits are not known. We test the hypothesis that chord progressions promote social bonding between dyads by shared temporal alignment of frequency spectra. Two musical conditions were presented to 20 pairs of participants (equal numbers of males and females), one with either a structured chord or predictable progression and the other with an unstructured and unpredictable composition of the same notes. Functional near-infrared spectroscopy signals were recorded simultaneously from both partners during the music conditions with and without gazing at a live partner's face. The right angular gyrus, right somatosensory association cortex, and bilateral dorsal lateral prefrontal cortex increased activation during live face gaze combined with the structured chord progression condition. Further, subjective ratings of subjective connectedness were associated with both activity in the right superior and middle temporal gyri during face gaze and the right angular gyrus during chord progressions. These findings link live face-to-face gaze while listening to structured chord progressions to neural systems that are responsive to predictive alignment of co-occurring acoustic spectra and perceptions of social connectedness.

## Significance Statement

Music is universally appreciated as a promoter of social bonding and a candidate for therapeutics for social disconnection syndromes. However, a theoretical framework and the necessary link between neural correlates of social behavior and specific features of music are not established. We test the hypothesis that listening to consonant chord progressions during live face gaze relative to corresponding scrambled notes promotes social bonding and activates social neural systems. Subjective ratings of social connectedness, neural activity observed in social systems, and cross-brain neural synchrony support the hypothesis that musical chord progressions are a salient musical feature that upregulates social neural systems. These findings advance an evidence-based framework for use of musical chord progressions to treat symptoms of social disconnection and isolation.

## Introduction

Social connections are critical for human health, survival, and solutions to loneliness. Nonetheless, social isolation and the related mental health conditions are understudied (Eisenberger and Cole, 2012; Kennedy and Adolphs, 2012; Allen et al., 2014). Here, we investigate the effects of music as a promoter of socialization. Music is widely considered a universal mediator of prosocial human-to-human interaction (Zatorre, 2005; Frith and Frith, 2012; Greenberg et al., 2021; Bigand and Tillmann, 2022) and has been associated with synchronization of emotions and actions that facilitate and reinforce prosocial relationships. For example, the prosocial effects of music on children have been associated with increased spontaneous cooperative play, helping behavior, and enhanced synchrony in contrast to non-music activities (Kirschner and Tomasello, 2010; Feldman et al., 2011; Kokal et al., 2011; Cirelli et al., 2014; Good and Russo, 2016; Kniffin et al., 2017). Group singing in adults increases perceived social closeness and bonding and lowers the pain threshold (Weinstein et al., 2016; Camlin et al., 2020). Further, synchronized musical activities, such as group drumming, enhance social cohesion (Gordon et al., 2020), while collaborative songwriting has been shown to enhance peer and social connectedness (Bourdaghs and Silverman, 2023; Perkins et al., 2023).

Knowledge of and familiarity with the music, shared goals and strategies, and various social factors have been associated with facilitation of interpersonal processes (Abalde et al., 2024). Importantly, familiarity of the music modulates large-scale cortical and subcortical networks that are activated during music listening (Vuong et al., 2023). Yet, despite the abundance of evidence for the impact of music on social behavior and the potential beneficial effects for reduction of anxiety, depression, and loneliness (Nilsson, 2008; Aalbers et al., 2017; Bradt et al., 2021; Chen et al., 2024), there remains little understanding of how specific features of music modulate social neural networks in the brain. Thus, the potential application of music as a therapeutic tool has been underdeveloped partially due to this paucity of evidence-based models for treatment applications.

The nature of music includes statistical universals that are present across cultures including isochronous beat and discrete pitches with nonequidistant scales (Savage et al., 2015; Mehr et al., 2019; McPherson et al., 2020; Yurdum et al., 2023). Using EEG and fMRI, characteristic signatures of auditory perception have been mapped for chord sequences to areas such as the auditory cortex and prefrontal cortex (Blood and Zatorre, 2001; Garza Villarreal et al., 2011). For example, when a chord is played that does not fulfill the expected harmonic progression, an event-related potential is evident in both non-musicians and musicians (Koelsch et al., 2007; Zhang et al., 2018; Pagès-Portabella and Toro, 2020), and unexpected chords in a sequence have been shown to modulate amygdala activity (Koelsch et al., 2008). It has been posited that chord progressions promote social bonding by reducing uncertainty through predictable temporal alignments of specific co-occurring acoustic spectra (Savage et al., 2021). Although it has been shown that music activates many brain regions involved in social networks such as the prefrontal cortex, anterior cingulate cortex, and amygdala (Blood et al., 1999; Janata et al., 2002; Gosselin et al., 2007; Koelsch et al., 2008; Bianco et al., 2022), there is little theoretical framework for a link between neural systems that underlie social behavior and specific features of music.

Here we test the hypothesis that musical chord progressions experienced during a dyadic social context (live face gaze) activate neural mechanisms known to be associated with social processes. We focus on the ii-V-I-vi progression, a common consonant chord progression encountered ubiquitously in jazz and other popular genres of Western music (Rosenberg, 2014; Miles et al., 2017; White and Quinn, 2018; Jimenez et al., 2020; Brown et al., 2021). Specifically, we hypothesize that the harmonic structure of predictable chord progressions upregulate prosocial systems in the brain relative to the effects of the same tones without chord progression.

## Materials and Methods

In the current study, we employed contrast comparisons of functional neural imaging data, cross-brain synchrony, and subjective reports of perceived social connectedness to isolate the effects of exposure to chord progressions common in Western popular music (Rosenberg, 2014; Miles et al., 2017; White and Quinn, 2018; Jimenez et al., 2020; Brown et al., 2021) relative to the control condition without chord progressions. Functional near-infrared spectroscopy (fNIRS) was employed to measure neural responses during live dyadic interactions. This neuroimaging technology uses optical methods that measure the absorption of wavelengths of light specific to oxyhemoglobin (OxyHb) and deoxyhemoglobin (deOxyHb) to identify the locations of active brain areas (Jöbsis, 1977; Villringer and Chance, 1997; Buxton, 2009; Ferrari and Quaresima, 2012; Scholkmann et al., 2014). The primary advantage of this imaging technology is that live interacting individuals can be imaged simultaneously in upright and more natural conditions while wearing head-mounted caps populated with small detectors and light emitters. This technology has been widely applied to investigations of social interactions and cognition (Cutini and Brigadoi, 2014; Ferreri et al., 2014; Hirsch et al., 2017, 2018, 2022, 2023; Yücel et al., 2017; Zhao and Cooper, 2017; Pinti et al., 2018; Wass et al., 2020; Czeszumski et al., 2022; Gugnowska et al., 2022). It has also been applied to live face-to-face communication during drumming using the dyadic fNIRS imaging system to activate social systems in the brain (Rojiani et al., 2018).

The terms “chord progression” and “no-chord progression” are used to describe our musical stimuli. Predictable and consonant musical motifs constructed with the ii-V-I-vi chord progression are the key features that are maintained across all stimuli in the “chord progression” condition. This progression follows a consonant and predictable tension and release that is typical in Western popular music. Importantly, in these experiments, the chord progression condition has a consonant, organized chord progression (ii-V-I-vi) played by instruments with a pleasing timbre, constant rhythm, and concordant temporal alignment with the drum beat. In contrast, the “no-chord progression” condition has the same number and range of different notes, timbre, and constant rhythm; however, the organization of the harmonic progression and the temporal structure of the piano and bass is disrupted while the temporal relation to the underlying drum structure remains unchanged. Both conditions were matched using the same notes, volume, and musical instruments. The presence of a rhythmic structure of a kick drum playing half notes and a ride cymbal playing quarter notes establishes a constant, discernible, and predictable rhythm across conditions. Varying degrees of syncopation can affect the perceived rhythmic stability in music and is dependent on degree of polyphony, instrumentation, and musical training (Weaver, 1939; Witek et al., 2014). Thus, while the drumbeat is the same across both conditions, the degree of perceived rhythmic concordance and complexity is different due to the highly syncopated and unpredictable nature of the piano and bass notes. Evidence in favor of the hypothesis that predictable chord progressions enhance social interaction would include an increase in chord progression-related neural activity relative to the no-chord progression condition during live face-to-face gaze which is employed as a form of social interaction.

The effect of chord progressions on subjective ratings of social bonding, i.e., connectedness to their partner, was evaluated by comparison of the ratings for each of the conditions. These measures of social connectedness were employed to test the hypothesis that social bonding is highest for the face-to-face with chord progression condition. This hypothesis is related to the proposed neural coupling hypothesis (Hasson et al., 2012; Hasson and Frith, 2016) suggesting that cross-brain neural synchrony reflects dynamically shared information between the interacting dyads including mutual face gaze and mutual predictability of the chord progressions. Together, live dyadic neuroimaging, neural coupling, and self-report measures of social connections were employed to compare neural representations of musical chord progression versus no-chord progression as well as face-to-face gaze versus no-face gaze.

Participants were adults (20 men, 18 women, 2 nonbinary; 37 right-handed, 3 left-handed) who were at least 18 years (mean age: 27.2 ± 9.6 years) and self-reported as typically healthy with no known neurological disorders. See Table 1. Participants were assigned to dyads based on their schedule and availability. Demographics information, baseline familiarity with their dyadic partner, and musical experience were also provided. Our target sample size of 20 dyads is based on power analyses detailed in previous dyadic and interactive investigations (Hirsch et al., 2022; Zhao et al., 2023) where it was determined that a sample of 15 dyads (*n* = 30) was sufficient to achieve a power of 0.80 in their investigation (Hirsch et al., 2023). In the current work, we targeted a sample size of 20 dyads to increase certainty in our analysis. All participants provided written informed consent in accordance with approved guidelines established by the Yale University Human Investigation Committee (HIC #1501015178) and were compensated for participation.

**Table 1.** Participant demographics and music engagement

Participants (*N* = 40)	
Age (average)	27.2 ± 9.6
Gender	
Female	18
Nonbinary	2
Male	20
Race	
Biracial	2
White	23
Asian	15
Ethnicity	
Latinx/Hispanic	4
Right-handed	37
Familiar with partner before study (yes)	11
Dyadic gender composition (*N* = 20)	
Female/female	4
Nonbinary/female	1
Nonbinary/male	1
Female/male	9
Male/male	5
Past or current music engagement^a^	
Engagement (yes)	29
≥7 years engagement	17
≥3 h/week current engagement	6

a Measurements were unavailable for two participants.

### Ethics statement

Ethical approval was obtained from the Yale University Human Research Protection Program [HIC # 1501015178, “Neural mechanisms of the social brain” (J.H.)]. Informed consent was obtained from each participant in accordance with established guidelines.

### Experimental setup and paradigm

**Figure 1.**
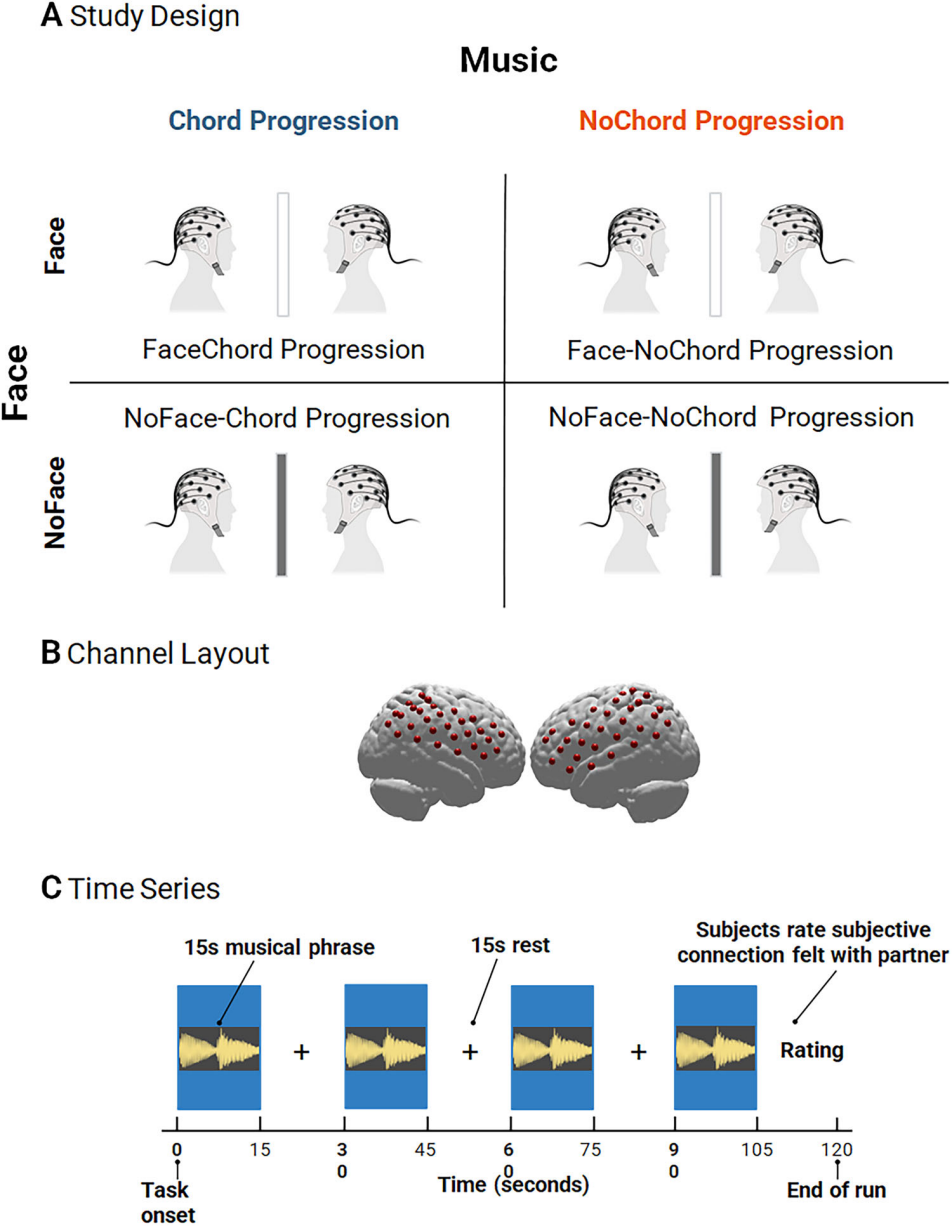
***A***, Illustrations of the two-person experimental setup and configuration for simultaneous fNIRS recording and face gaze conditions. A “smart-glass” bisects person-to-person distance. Face and NoFace conditions are represented by transparent and opaque dark gray “smart-glass” bars, respectively. ***B***, Right and left hemispheres of a single rendered brain to illustrate median channel locations (red dots) for 29 channels per participant. ***C***, Time series for a single run.

Dyadic participants were positioned 140 cm across a table from each other with a custom-made and controllable “smart glass” window that toggled between transparency and opacity according to the experimental time series. Each participant wore a cap with an array of “optodes” (small detectors and emitters for the acquisition of hemodynamic signals) that provided coverage over both hemispheres of both participants, specifically targeting bilateral temporoparietal junction and inferior frontal and middle gyri (Fig. 1*A*,*B*). This arrangement allowed for simultaneous recording of two individuals while acquiring fNIRS signals.

The experimental design consisted of two factors: live face gaze and chord progression with two levels on each: face and no-face, and chord progression and no-chord progression. The timing of the four conditions was controlled by the smart glass that alternated between clear and opaque and paradigm controls that presented the musical conditions. The four conditions were (1) live face-chord progression, (2) no-face-chord progression, (3) live face-no-chord progression, and (4) no-face-no-chord progression. Each run consisted of four 15 s task periods separated by rest blocks (15 s). Each run was administered twice, totaling eight runs (duration: 16 min). Each consisted of four 15 s stimuli separated by rest blocks (15 s; Fig. 1*C*). During each live face condition, the smart glass became transparent so that participants had a full view of their partner's face and could view each other freely. During no-face conditions and rest blocks between stimuli, the smart glass was opaque, and participants could not see their partner's face. Participants were instructed to view the transparent or opaque glass during each stimulus and rest period.

### Instructions to participants

Participants were instructed to gaze naturally at the face of their partner during the time periods when the smart glass was clear. Natural facial expressions and eye contact were encouraged. However, talking was not permitted, and participants were advised to avoid excessive head movement, deep breathing, yawning, and face touching.

### Connectedness ratings

After each run, participants were asked to indicate “How connected do you feel to your partner?” using a computer monitor located above of the smart glass and a dial to display integers ranging from 0 to 5 indicating neutral to very connected, respectively. A response of “0” was considered to be a nonanswer. As a baseline recording, participants were also asked to rate subjective connectedness after meeting during the consent process but before entering the experimental room. Participants were instructed to rate connectedness based on their own understanding of connection. If participants had further questions about how they should rate the connection they felt with their partner, we emphasized that there was no right or wrong answer and that their ratings would not be disclosed to their partner. Some of the participants (60%) were asked to retroactively rate connectedness felt before the start of the experiment.

Responses were averaged by condition across participants and differences were assessed using a Kruskal–Wallis test and Games–Howell post hoc *t* tests because the assumption of homogeneity of variance required in parametric tests could not be validated. Results were confirmed by pairwise *t* test with Bonferroni’s correction (Kassambara, 2023) in R.

### Creation of stimuli and listening equipment

Chord progressions are a feature of most Western popular music and are a mathematically defined set of frequencies with a specific temporal relationship that provides structure and context to a musical composition (Zatorre and Salimpoor, 2013; McPherson et al., 2020; Weiss et al., 2020; Nguyen et al., 2023). Perception and preferences for consonant chord progressions, a sequence of chords that are primarily composed of intervals considered harmonious and pleasing, emerges within infants, suggesting its fundamental importance in music perception (Schellenberg and Trainor, 1996; Zentner, 1996; Trainor, 1997). Chord progressions for this experiment were prerecorded by the experimentalists. They were created in Logic Pro X with a Yamaha MODX6 61-key synthesizer. Two stimulus sets were created, a “chord progression” and a “no chord progression” set. Examples of each type of stimulus set can be heard at the following link: https://on.soundcloud.com/Q7EJ7B7XtRrbbhxW6. Images of Musical Information Digital Interface (MIDI) information and musical scores of example stimuli in the key of E major can be found in Figure S1.

Each chord progression stimulus set was designed to have a predictable, consonant chord progression played by instruments with a pleasing timbre with constant rhythm and dynamics throughout the stimulus (Dvorak and Hernandez-Ruiz, 2021). We used the ii-V-I-vi chord progression as it is a common harmonic progression in Western music encountered with high prevalence in popular music across genres (Rosenberg, 2014; Miles et al., 2017; Brown et al., 2021). Each chord progression stimulus had a set tempo of 140 beats per minute, lasted 15 s, and consisted of four tracks:

1. Track 1: a piano playing a simple nonsyncopated melody with half notes derived from the pentatonic scale of the key outlined by tracks 2 and 3.
2. Track 2: a piano playing a ii-V-I-vi harmonic progression with half note chords ending on the tonic (I) of the key. For example, in the key of C major the chords were D minor 7 (ii), G dominant 7 (V), C major 7 (I), and A minor 7 (vi). This progression was repeated three times and then resolved on the C major 7 (I) on the fourth cycle.
3. Track 3: a bass playing quarter notes that outlined a ii-V-I-vi chord progression that accompanied track 2. For example, in the key of C the bass would play a D (ii), then G (V), then C (I), followed by A (vi).
4. Track 4: a drum pattern with a ride cymbal playing quarter notes and a kick drum playing half notes.

Each exemplar in the chord progression set used the same chord progression (ii-V-I-vi) and chord voicings; however, each was created in 1 of the 12 different keys. One of four possible consonant melodies derived from the pentatonic scale were randomly assigned to each stimulus across the 12 keys. This resulted in 12 novel stimuli with a shared consonant harmonic and rhythmic structure that served as the chord progression stimuli. The control set of musical stimuli was generated by temporally (250 ms–2 s) shuffling the notes of the tonal instruments in tracks 1–3 (piano and bass) while leaving track 4 (drums) unaltered. This resulted in disruption of the harmonic and rhythmic context for the tonal component of the nonharmonic stimuli while leaving the rhythmic pattern and tempo information of the drums intact. All tracks were mixed, and sound levels adjusted in Logic Pro X. Participants listened to the stimuli through JBL Control 1 Pro Two-Way Professional Compact Loudspeakers. Each participant had a set of two speakers angled toward them so that the stimuli converged at ear level. Acoustic features were extracted from all 24 soundtracks (12 chord progression, CP, and 12 no-chord progression, NCP, using MIRToolbox; Lartillot and Toiviainen, 2007) and averaged to compare physical characteristics within and between conditions (Lartillot et al., 2008; Lee et al., 2023; Cheung et al., 2025) See Table 2.

**Table 2.** Acoustic features

Feature	Definition	Chord progression	No-chord progression	*t* test (CP > NCP)
		Mean (±SD)	Mean (±SD)	
Rhythm				
Tempo	Beats per minute of rhythmic grid	140 BPM	140 BPM	N/A
Peak magnitude (fluctuation spectrum, a.u.)	Amplitude of the dominant modulation frequency in the fluctuation spectrum	2,135.00 (115.71)	1,240.53 (202.63)	*t*_(22)_ = 13.28, *p* < 10^−11^
Peak-to-median ratio	Salience of dominant rhythmic modulation relative to background modulation energy	11.68 (0.70)	5.67 (0.91)	*t*_(22)_ = 18.09, *p* < 10^−13^
Pulse clarity (a.u.)	Strength and regularity of perceived periodic beat structure	0.33 (0.07)	0.48 (0.09)	*t*_(22)_ = −4.60, *p* < 10^−2^
Fluctuation-spectrum entropy	Spectral flatness of modulation energy across rhythmic frequencies	0.95 (0.00)	0.98 (0.00)	*t*_(22)_ = −24.11, *p* < 10^−16^
Timbre				
Spectral flux (normalized)	Frame-to-frame spectral change reflecting timbral dynamics	2.1 × 10^−3^ (0.00)	2.6 × 10^−3^ (0.00)	*t*_(22)_ = 6.50, *p* < 10^−4^
Root-mean-square (RMS; dBFS)	Overall signal energy averaged across time	−19.46	−22.56 (0.00)	*t*_(22)_ = −4.60, *p* < 10^−5^
Spectral centroid/brightness (Hz)	Mean frequency weighted by spectral energy	905.45 (36.51)	942.19 (31.27)	*t*_(22)_ = −2.65, *p* = 0.01

Spectral flux, centroid, and RMS were computed on mono-summed audio using short-time Fourier analysis (46 ms windows, 10 ms hop). Fluctuation-spectrum measures were derived from modulation spectra computed using MIRtoolbox (Lartillot and Toiviainen, 2007). a.u., arbitrary units; dBFS, decibels relative to full scale.

### Acoustic characteristics

The comparison of acoustic characteristics related to rhythm and timbre for each of the two musical conditions is included in Table 2. Rhythm is compared on features including tempo, peak magnitude, peak-to-median ratio, pulse clarity, and fluctuation-spectrum entropy; and timbre is compared on spectral flux, root-mean-square, and spectral centroid/brightness. Each is defined in column 2 of Table 2. Mean and standard deviation for each condition (chord progression and no-chord progression) based on the 12 unique exemplars of each are shown in the middle columns. The right-hand column indicates the results of the statistical comparison of [chord progression (CP) >no-chord progression (NCP)]. In summary, for the rhythm features where tempo was constant between the two conditions, both peak magnitude (*p* < 10^−11^) and peak-to-median ratio (*p* < 10^−13^) were greater for the chord progression conditions, whereas the pulse clarity (*p* < 10^−2^) and fluctuation-spectrum entropy (*p* < 10^−16^) were greater for the no-chord progression condition. For the timbre features, spectral flux was greater for the chord progression condition (*p* < 10^−4^), whereas root-mean-square, dBFS, (*p* < 10^−5^) and centroid/brightness, Hz, (*p* < 0.01) were both greater for the no-chord progression condition. Overall, the conditions containing a structured chord progression exhibited stronger low-frequency modulation dominance, whereas the condition lacking harmonic structure exhibited greater rhythmic salience and spectral irregularity.

The observed differences in Music Information Retrieval, MIR, features between harmonic and nonharmonic conditions are consistent with established models of hierarchical musical organization (Koelsch et al., 2013; Mehr, 2025). The conditions containing structured chord progressions exhibited higher peak magnitude and peak-to-median ratios in the fluctuation spectrum, indicating the presence of dominant low-frequency modulation components and greater hierarchical coherence. These features have been associated with perceptual grouping, tonal expectation, and the integration of musical events over longer temporal windows. In contrast, stimuli in which tonal notes were shuffled while rhythmic elements were preserved showed higher pulse clarity and fluctuation spectrum entropy, reflecting increased salience of surface-level rhythmic periodicity and a more distributed modulation energy profile.

### Music experience and dyadic partner familiarity questionnaires

Surveys (Text S1) assessing familiarity of participants with their partner were administered following the experimental session. If participants responded affirmatively to knowing their partner prior to the day of the study, data on the duration and nature of their relationship was obtained. A 1–5 Likert scale similar to that which was used during the experiment was used by participants to indicate subjective connectedness. Participants were also asked to answer survey questions about any previous and current engagement with music (Fig. S2). Music engagement was any involvement including, but not limited to, playing or studying music theory, instrumental, or vocal techniques.

### Functional NIRS signal acquisition and channel localization

Functional NIRS signal acquisition, optode localization, and signal processing, including global mean removal, were similar to methods described previously (Kirilina et al., 2013; Zhang et al., 2016) and are briefly summarized below. Hemodynamic signals were acquired using three wavelengths of light (780, 805, and 830 nm), and an 80-fiber multichannel, continuous-wave fNIRS system (LABNIRS, Shimadzu). Differential absorption of each wavelength of light was converted to concentration changes for deOxyHb, OxyHb, and total combined deOxyHb and OxyHb using standard methods previously described (Matcher et al., 1995).

Each participant was fit with an optode cap with predefined channel distances. Three sizes of caps were used based on the circumference of the participants’ heads (60, 56.5, or 54.5 cm). Optode distances of 3 cm were designed for the 60 cm cap but were scaled equally to smaller caps. A lighted fiber-optic probe (Daiso) was used to displace all hair from the optode holder before optode placement. Optodes consisting of 20 emitters and 20 detectors were arranged in a custom matrix providing a total of 29 acquisition channels per participant. For consistency, the placement of the most anterior midline optode holder on the cap was centered 1 cm above nasion. To ensure acceptable signal-to-noise ratios, intensity was measured for each channel before recording, and adjustments were made for each channel until the optodes were calibrated and able to sense known quantities of light from each laser wavelength (Noah et al., 2015). Anatomical locations of optodes in relation to standard head landmarks were determined for each participant using a structure.io 3D scanner (Occipital) and portions of code from the FieldTrip toolbox implemented in Matlab 2022a (Eggebrecht et al., 2012; Homölle and Oostenveld, 2019). Optode locations were used to calculate positions of recording channels (Fig. 1*B*), and Montreal Neurological Institute (MNI) coordinates (Mazziotta et al., 2001) for each channel were obtained with NIRS-SPM software (Ye et al., 2009) and WFU PickAtlas (Maldjian et al., 2003, 2004).

### Signal processing

Raw optical density variations in the fNIRS signals were acquired at three wavelengths of light (780, 805, and 830 nm), which were translated into relative chromophore concentrations using a Beer–Lambert equation (Matcher et al., 1995). Signals were recorded at 30 Hz. Baseline drift was removed using wavelet detrending provided in NIRS-SPM (Ye et al., 2009). In accordance with recommendations for best practices using fNIRS data (Yücel et al., 2021), global components attributable to blood pressure and other systemic effects (Tachtsidis and Scholkmann, 2016) were removed using a principal component analysis (PCA) spatial global mean filter (Zhang et al., 2016, 2017; Hirsch et al., 2018; Noah et al., 2021) before general linear model (GLM) analysis. The HbDiff signal which is derived from the sum of the OxyHb and deOxyHb signals for all statistical analyses was utilized to optimize signal reliability (Kaynezhad et al., 2023). Following best practices (Yücel et al., 2021), baseline activity measures of both OxyHb and deOxyHb signals were processed as a confirmatory measure. The HbDiff signal averages are taken as the input to the second level (group) analysis (Tachtsidis et al., 2009). Comparisons between conditions were based on GLM procedures using NIRS-SPM (Ye et al., 2009). Event epochs within the time series were convolved with the hemodynamic response function provided from SPM8 (Penny et al., 2011) and fit to the signals, providing individual “beta values” for each participant across conditions. Group results based on these beta values were rendered on a standard MNI brain template (TD-ICBM152 T1 MRI template; Mazziotta et al., 2001) in SPM8 using NIRS-SPM software with WFU PickAtlas (Maldjian et al., 2003, 2004).

### General linear model analysis

The primary GLM analysis employed a standard block design. For each 2 min run, there were four blocks of 15 s of task each separated by four 15 s periods of rest and was implemented in NIRS-SPM (Ye et al., 2009), which uses SPM8 (Penny et al., 2011) inside MATLAB 2022.

### Neural coupling (coherence)

Cross-brain synchrony (neural coherence) was evaluated using wavelet analysis (Torrence and Compo, 1998; Zhang et al., 2020), as previously described (Hirsch et al., 2018). The wavelet kernel was a complex Gaussian provided by MATLAB. The number of octaves was 4 and the range of frequencies was 0.4–0.025 Hz, which is sensitive to the hemodynamic response function. The number of voices per octave was also 4, and therefore 16 scales were used for which the wavelength difference was 2.5 s. Methodological details and validation of this technique have been previously described (Zhang et al., 2017). The analysis was conducted by using concatenated segments collected during the experiment. This approach provided a measurement of nonsymmetric coupled dynamics (Hasson and Frith, 2016), where one participant's neural signals were synchronized with their partner's neural signals representing predictable transformations between the two brains. Signals acquired from predefined anatomical regions from visual cortex and parietal and temporal lobes were decomposed into temporal frequencies that were correlated across the two brains for each dyad following removal of the task regressor as is conventional for psychophysiological interaction analysis (Friston et al., 1997). Here we apply the residual signal to investigate effects other than the main task-induced effect. For example, cross-brain coherence of multiple signal components (wavelets) is thought to provide an indication of dynamic coupling processes rather than task-specific processes. Coherence during social music listening was compared for the four conditions: face-chord progression, face-no-chord progression, no-face-chord progression, and no-face-no-chord progression. This analysis was also applied to a comparison of “scrambled” (shuffled) pairs of participants. In this analysis, the neural data from participants who were not actually paired during the experiment was aligned to the music conditions for analysis. This control analysis was to confirm that the reported coherence was specific to the live dyadic interaction and not the possible effect of common processes across all the conditions.

## Results

### Behavioral measures of social connectedness

**Figure 2.**
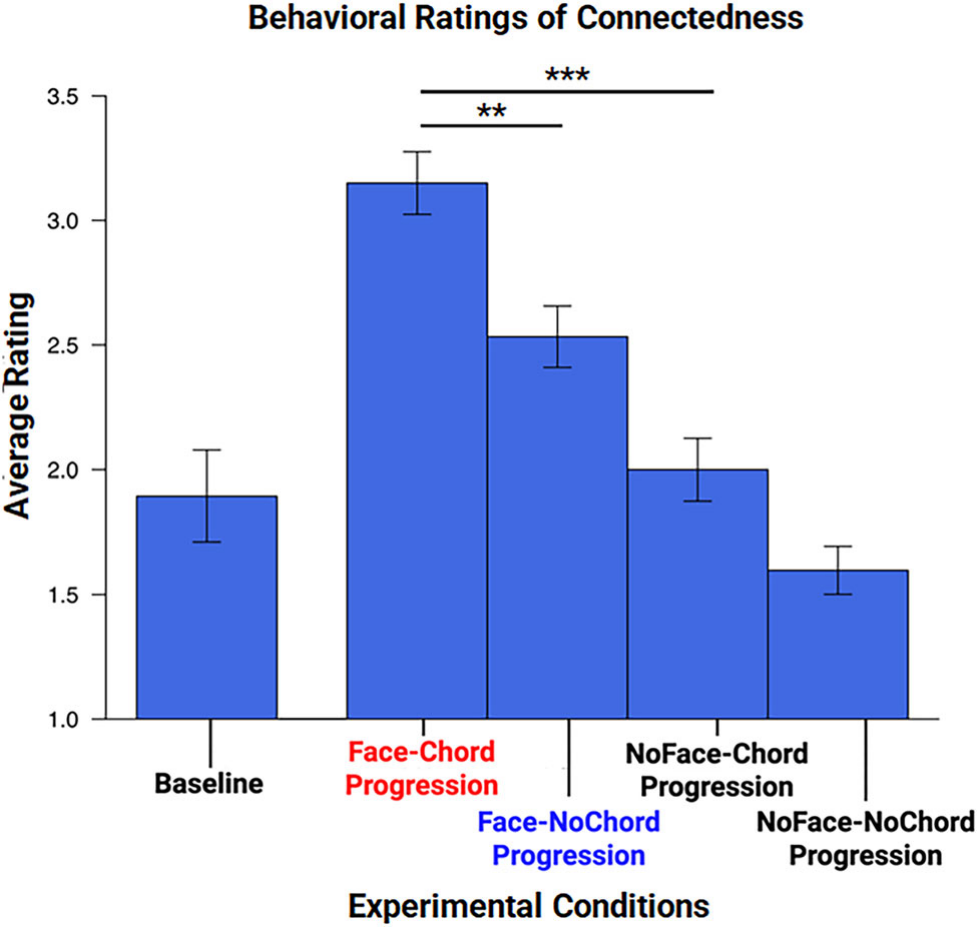
Average subjective ratings of connectedness are shown for each condition. The average rating of subjective connectedness was dependent upon the experimental condition [*χ*^2^ (4, *N* = 40) = 79.97, *p* < 0.001; *F*_(4,190)_ = 16.35, *p* < 0.001]. The highest average rating of connectedness was observed during the face-to-face gaze paired with the intact chord progression and the lowest average rating of connectedness was observed during the NoFace condition paired with the music without chord progression (NoChord progression). Baseline measures were acquired prior to the experiment. Twenty-four (60%) participants retroactively rated their baseline connectedness.

Average ratings of connectedness are shown on the *y*-axis of Figure 2 for each of the four conditions and the baseline acquired prior to the experiment. Participants reported the highest levels of connectedness during the face-chord progression condition when compared with all other conditions. No change in subjective connectedness was observed between baseline measurements and the no-face-chord progression or no-face-no-chord progression conditions. The findings are consistent with the hypothesis that musical chord progression is associated with perceptions of increased social connectedness during live social interactions such as live face-to-face gaze.

### Neural measures of face and chord progression conditions

**Figure 3.**
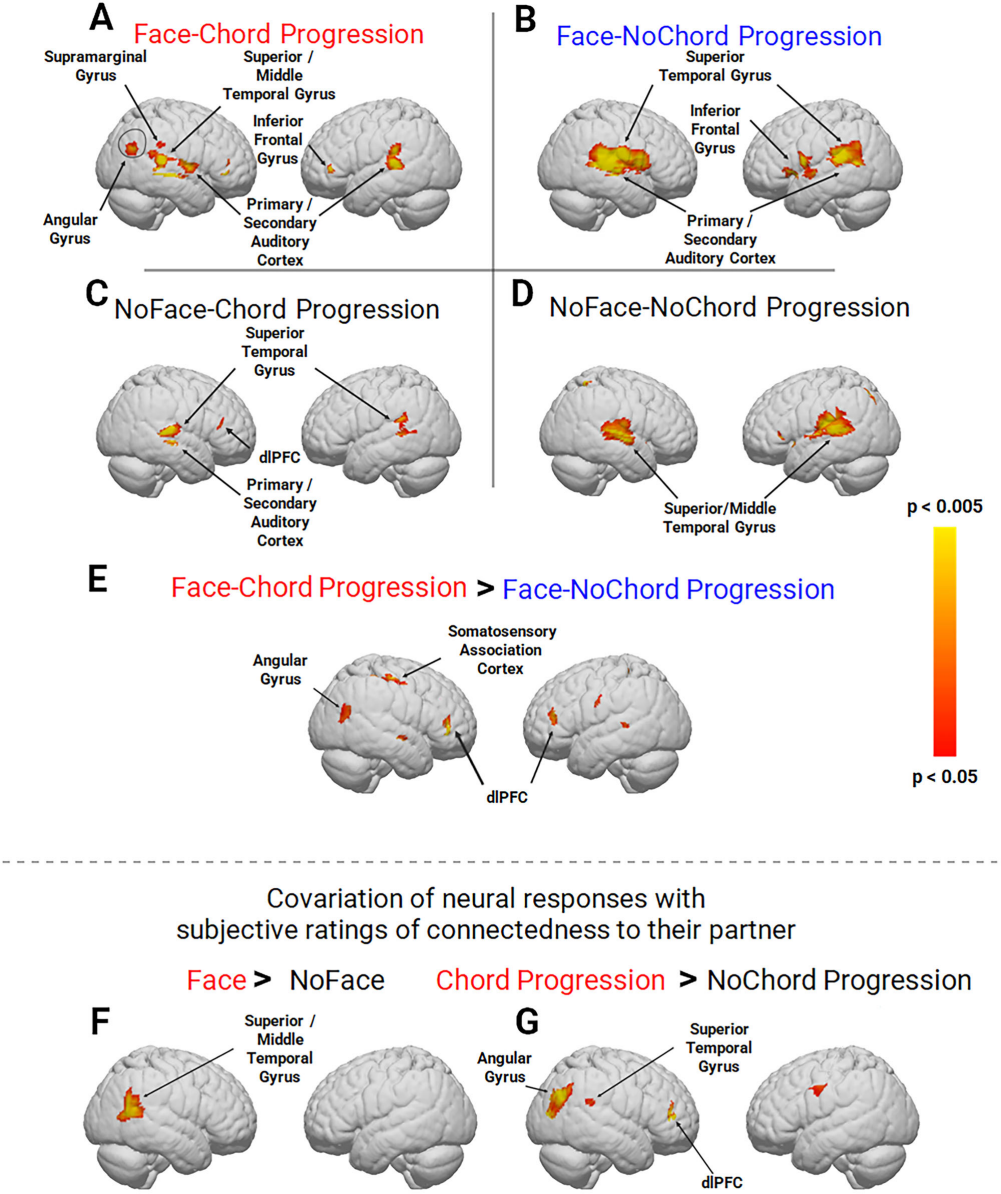
Average neural activation elicited by the following conditions: ***A***, Face-chord progression; ***B***, face-no-chord progression; ***C***, no-face-chord progression; ***D***, no-face-no-chord progression; and ***E***, face-chord progression > face no-chord progression. Panels ***A–D*** show the main effects for face (rows) and chord progression (columns). Panel ***E*** compares the two face conditions (with and without chord progression) and shows increased activity for the face-chord progression condition in right angular gyrus and primary somatosensory cortex in addition to frontal dLPFC activity (*p* < 0.05). Panels ***F*** and ***G*** show the neural activity that is correlated with the subjective ratings of connectedness to the partner. The right superior and middle temporal gyri are correlated with the face contrast (***F***) and the angular gyrus is correlated with the chord progression contrast (***G***; *p* < 0.05). See Table 3, A–G.

Main effects for each of the four conditions are shown on Figure 3*A–D*. Consistent with the hypothesis that chord progressions upregulate neural systems sensitive to social functions, the face-chord progression condition (*A*) shows an increase in the right hemisphere angular gyrus. The right angular gyrus has previously been associated with live face processing (Noah et al., 2021; Hirsch et al., 2023) consistent with the hypothesis that live face processes are amplified under conditions of shared listening to music with chord progression relative to shared listening to the same auditory stimuli without chord progression. Interestingly, the activity clusters observed during the no-chord progression conditions [either with a live face interaction (*B*) or without (*D*)] are larger than clusters with chord progression suggesting an increased sensory response to the “more complex” and uncommon auditory condition. The comparison of the two face conditions, face paired with chord progression and face paired with the no-chord progression (face-chord progression > face-no-chord progression; *E*), showed increased engagement of right angular gyrus as well as right somatosensory cortex and bilateral DLPFC (*p* < 0.05). These observations are consistent with the hypothesis that live face-to-face interaction and musical chord progression cooperate to amplify neural activity in these specific regions.

### Neural correlates of connectedness

Neural correlates of perceived social connectedness are shown in Figure 3*F*,*G*. The relationship between the ratings of connectedness and neural activity was determined for the two experimental factors: face and chord. Individual ratings following each run were applied as covariates on the neural responses prior to the contrast comparisons (*p* < 0.05). Consistent with well-known face processing specializations, the face-no-face contrast (*F*) shows an activity cluster in the right superior and middle temporal gyri consistent with a “neural correlate” coassociated with social connectedness and live face gaze. Consistent with the main effects of this study that compare chord progression and no-chord progression conditions (*G*), the right angular gyrus cluster (including the superior temporal gyrus and the DLPFC) is active and suggests a coassociation with the ratings of connectedness and the chord progression condition (*p* < 0.05). Table 3 identifies cluster locations, anatomical labels, Brodmann's area (BA), and probabilities for regions observed for the conditions and analyses shown in Figure 3*A–G*.

**Table 3.** Functional and anatomical results corresponding to Figure 3

MNI coordinates^a^			*p*	*t*	df	Anatomical region	BA	Probability
*X*	*Y*	*Z*						
A. Condition—face-chord progression (Fig. 3*A*)								
72	−25	−3	0.0016	4.14	8	MTG	21	0.62
						STG	22	0.34
67	−1	3	0.0069	2.63	27	STG	22	0.63
−55	40	3	0.0022	3.58	11	Inferior frontal G	47	0.42
						dlPFC	46	0.25
						Pars triangularis	45	0.25
72	−33	11	0.0005	3.66	29	STG	22	0.64
						Primary and auditory association cortex	42	0.35
−70	−31	9	0.0019	3.2	24	STG	22	0.51
						Primary and secondary auditory association cortex	42	0.38
60	−63	25	0.0084	2.51	35	Angular gyrus	39	0.71
70	−35	29	0.0264	2	39	Supramarginal gyrus	40	0.9
B. Condition—face-no chord progression (Fig. 3*B*)								
−64	7	13	0.0033	2.89	35	Pars opercularis	44	0.44
						Premotor and supplementary motor cortex	6	0.4
−53	15	−2	0.0043	3.09	13	STG	22	0.34
						Inferior frontal G	47	0.31
72	−29	11	0.0001	4.34	29	Primary and secondary auditory association cortex	42	0.49
						STG	22	0.47
−70	−37	11	0.0018	3.14	31	STG	22	0.65
C. Condition—no-face-chord progression (Fig. 3*C*)								
−68	−39	23	0.0069	2.59	36	Supramarginal gyrus	40	0.58
						STG	22	0.38
72	−29	7	0.0012	3.4	24	STG	22	0.56
						Primary and secondary auditory association cortex	42	0.35
58	35	15	0.0193	2.15	33	dlPFC	46	0.65
						Pars triangularis	45	0.35
D. Condition—no-face-no chord progression (Fig. 3*D*)								
70	−11	−1	0.0029	3.18	16	MTG	21	0.58
						STG	22	0.32
−70	−39	9	0.0023	3.15	22	STG	22	0.88
−56	29	1	0.0088	2.58	21	Pars triangularis	45	0.39
−45	−69	52	0.0061	2.87	14	Somatosensory association cortex	7	0.55
						Supramarginal gyrus	40	0.26
49	15	−4	0.0291	2.21	8	Inferior frontal G	47	0.47
						Temporopolar area	38	0.39
41	−54	63	0.0052	3.68	6	Somatosensory association cortex	7	0.48
						Supramarginal gyrus	40	0.34
E. Condition—face-chord progression > face-no chord progression (Fig. 3*E*)								
70	−11	−7	0.0216	2.4	8	MTG	21	0.94
55	44	1	0.0073	4.12	4	Inferior prefrontal gyrus	47	0.48
−70	−35	7	0.0195	2.22	19	STG	22	0.63
−51	41	19	0.0077	2.59	27	dlPFC	46	0.94
61	−62	25	0.0152	2.26	35	Angular gyrus	39	0.66
−65	−5	33	0.0207	2.11	37	Premotor and supplementary motor cortex	6	0.82
56	−17	57	0.005	2.73	34	Primary somatosensory cortex	3	0.48
−39	−39	61	0.0124	2.56	12	Primary somatosensory cortex	2	0.41
						Supramarginal gyrus	40	0.32
F. Condition—face > no face: covariation of neural responses with ratings of social connectedness (Fig. 3*F*)								
63	−60	11	0.054	2.76	39	MTG STG	21/22	0.96
G. Condition—chord progression > no chord progression: covariation of neural responses with ratings of social connectedness (Fig. 3*G*)								
61	−63	25	0.0011	6.78	39	Angular gyrus/V3	39	0.97
51	−81	8.4	0.012	6.78	39		19	
70	−37	25	0.025	2.03	39	STG	22	0.39
52	47	9	0.012	9.66	39	dLPFC	46	0.57
						Pars triangularis	45	0.43

^a^*X,Y,Z*, coordinates of centroid clusters are based on the MNI system (Mazziotta et al., 2001). − indicates left hemisphere; *p*, probability; *t* statistic and, df, degrees of freedom; BA, probability refers to the likelihood that the named region is correct. BA, Brodmann’s area; MTG, middle temporal gyrus, STG, superior temporal gyrus; dlPFC, dorsolateral prefrontal cortex.

### Neural coupling

**Figure 4.**
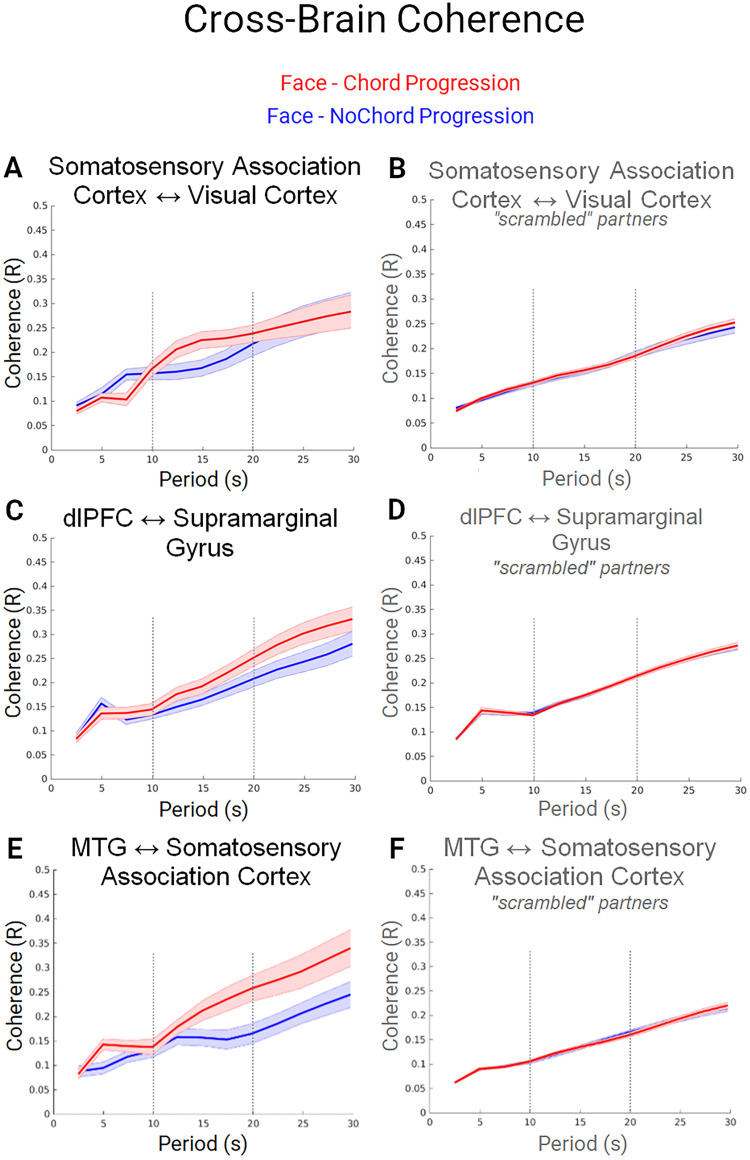
Dyadic cross-brain neural coherence for the face conditions. Cross-brain coherence of the neural signals was observed between the somatosensory association cortices and the visual cortex, ***A***, ***B***; the DLPFC and the supramarginal gyrus, ***C***, ***D***; and the middle temporal gyrus and the somatosensory association cortex, ***E***, ***F***. Signal coherence between participants (*y*-axis) is plotted against the period of the frequency components (*x*-axis) for the face condition paired with chord progression (red) and the face condition paired with the no-chord progression (blue) conditions. Solid lines represent mean data and shading represents standard error: the left panel shows that the coherence between the partners is increased (***A***, *t*_(28)_ = 2.96, *p* < 0.006; ***C***, *t*_(37)_ = 2.72, *p* < 0.01; ***E***, *t*_(21)_ = 2.99, *p* < 0.001) during the chord progression condition between wavelets between 10 and 20 s. The right panels (***B***, ***D***, ***F***) show the coherence between the scrambled (shuffled) partners and serve as a control for the effects of partner specific interaction. There is no evidence in favor of the coherence effect when the partners are scrambled in support of the interpretation that the effects represent partner-specific and social interactive reactions.

**Figure 5.**
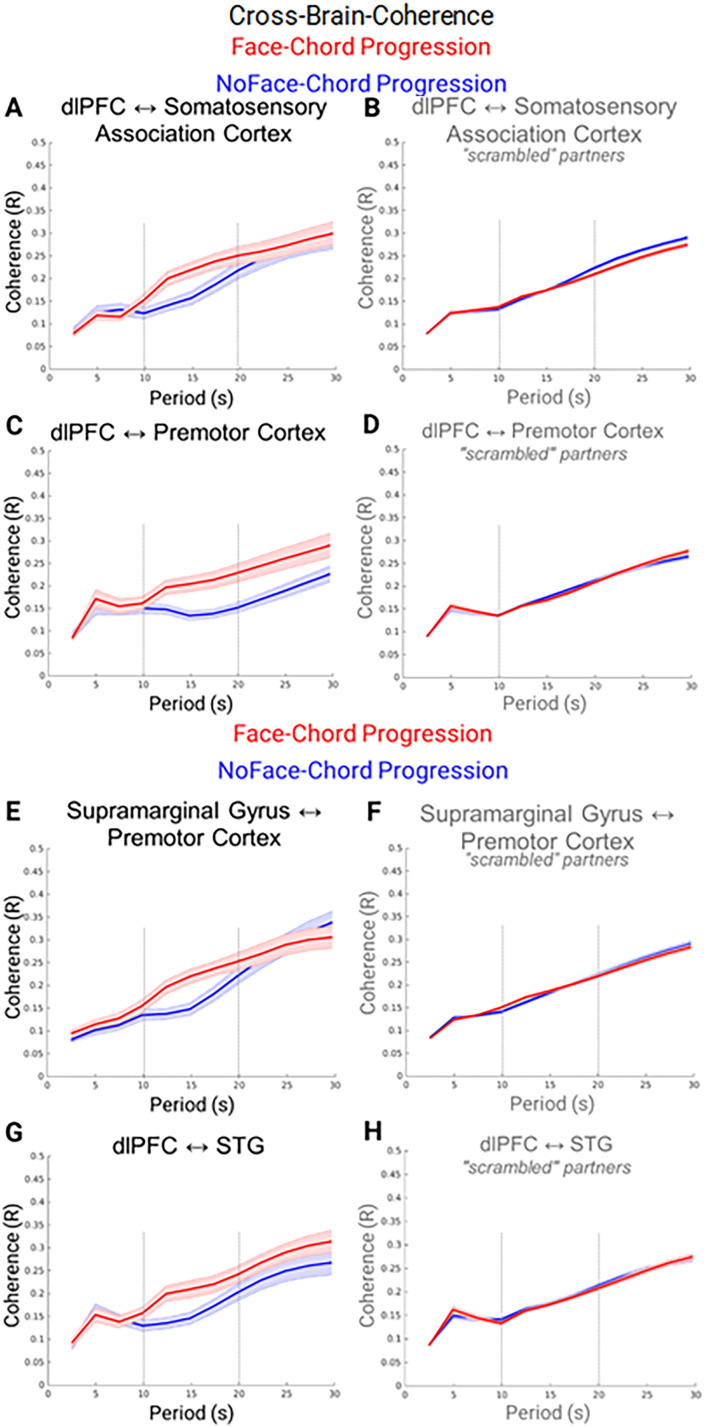
Dyadic cross-brain neural coherence for the chord conditions. Cross-brain coherence of the neural signals was observed between the DLPFC and the somatosensory association cortices, ***A***, ***B***; the DLPFC and the premotor cortex, ***C***, ***D***; the supramarginal gyrus and premotor cortex, ***E***, ***F***; and the DLPFC and the superior temporal gyrus, ***G***, ***H***. Signal coherence between participants (*y*-axis) is plotted against the period of the frequency components (*x*-axis) for the chord progression paired with the live face (red) and the chord progression paired with the NoFace (blue) conditions. Solid lines represent mean data and shading represents standard error: the left panel shows that the coherence between the partners is increased (***A***, *t*_(37)_ = 3.14, *p* < 0.003; ***C***, *t*_(37)_ = 3.44, *p* < 0.001; ***E***, *t*_(39)_ = 3.87, *p* < 0.001; ***G***, *t*_(37)_ = 3.14, *p* < 0.003) during the chord progression condition between wavelets between 10 and 20 s. The right panels (***B***, ***D***, ***F***, ***H***) show the coherence between the scrambled (shuffled) partners and serve as a control for the effects of partner specific interaction. There is no evidence in favor of the coherence effect when the partners are scrambled in support of the interpretation that the effects represent partner-specific and social interactive reactions.

According to the neural coupling hypothesis (Hasson et al., 2012), we expect to observe cross-brain neural coupling between regions that are most active during the live social interaction where information is shared between interacting participants. This subset of hypothesized regions includes angular gyrus, somatosensory association cortex, DLPFC, superior and middle temporal gyri, supramarginal gyrus, and premotor cortex (Hirsch et al., 2017; Noah et al., 2020). Neural coupling is considered for each and for the two experimental factors: face (Fig. 4) and chord (Fig. 5). For both figures, the cross-brain neural coherence (*y*-axis) shows the correlation of corresponding frequency components (wavelets; *x*-axis) of the neural responses across interacting partners. The *x*-axis represents the continuum of the frequency components in wavelets. The range of wavelengths shorter than 30 s was included since the experimental cycle including task and rest periods was 30 s (Fig. 1*C*).

A comparison of “scrambled” (shuffled) pairs of participants was also conducted as a control analysis to confirm that the reported coherence was specific to the live dyadic interaction and not due to engagement in a similar task (Figs. 4, 5, right column). In this comparison the two participants were not real interacting partners. There was no evidence for a difference in coherence between the two nonreal partner conditions suggesting that the increase in cross-brain coherence for the face-chord progression condition (left panel) is related to the live and reciprocal sharing of subtle social and visual cues such as facial expression and eye contact between the dyads.

In Figure 5, the chord progression-face condition (red line) is associated with greater neural coupling than the chord progression-no-face condition (blue line) for the DLPFC and somatosensory association cortex (Fig. 5*A*); the DLPFC and the premotor cortex (Fig. 5*C*); the supramarginal gyrus and the premotor cortex (Fig. 5*E*); and the DLPFC and the superior temporal gyrus (Fig. 5*G*). The neural coherence between these regions was increased for wavelets with periods between 10 and 20 s during the chord progressions that were paired with the live face-to-face gaze (red lines) relative to the chord progressions that were paired with the no-face condition (blue lines). The 10–20 s period range is consistent with the hemodynamic time constant and suggests a physiological difference in the signal. A comparison of “scrambled” (shuffled) pairs of participants was also conducted to confirm that the reported coherence was specific to the live dyadic interaction (Figs. 4, 5, right column). In this comparison the two participants were not the real interacting partners, and there was no evidence for a difference in coherence between the two conditions. This finding suggests that the increase in cross-brain coherence is related to the live and reciprocal sharing of subtle social cues.

## Discussion

The beneficial effects of shared music are generally considered to be universal. However, the underlying neural mechanisms for the social benefits remain understudied without an organizing theoretical framework. We address this knowledge gap by testing the hypothesis that structured and predictable musical chord progressions facilitate neural mechanisms associated with both social and perceptions of social connectedness. Live face-to-face gaze was applied as the social interaction. Neural effects were compared using fNIRS hyperscanning while partners listened to musical clips composed with and without chord progressions. Consistent with our hypothesis, the right angular gyrus, supramarginal gyrus, superior/middle temporal gyri, and the auditory cortices, i.e., components of the social system, were most activated during live face gaze and chord progression conditions.

Given the role of the auditory cortex in processing music regardless of chord structure, we expect and see activation in the auditory cortex in all conditions whether there is a chord progression or live face. The auditory cortex has a primary role in processing higher-order auditory information and has been implicated in processing the consonance of chord progressions (Daikoku et al., 2012; Cheung et al., 2019). Of the regions associated with social processing, the angular gyrus (AG) was observed only during live-face and chord progression. The AG is a hub for multimodal processing that integrates semantic information for comprehension, regulates attention, and links perception to action (Seghier, 2013; Tanaka and Kirino, 2019; Song et al., 2023). The AG has been associated with thematic relations and predictions based on its wide connections and structural heterogeneity (Davis and Yee, 2019; Farahibozorg et al., 2022). Interestingly, AG has also been implicated in schizophrenia, depression, bipolar, and social anxiety (Yüksel et al., 2018; Zeng et al., 2021; Picó-Pérez et al., 2022) and has been correlated to music-based analgesia (Garza-Villarreal et al., 2017). In this study, the AG was activated only during social interaction paired with the common consonant chord progression. Thus, these findings suggest that the angular gyrus may be a unique hub for the intersection of social systems and musical features with predictable progressions (Bravo et al., 2017).

In addition to neural systems supporting social and perceptual systems within brains, we consider the cross-brain systems. Neural activity within a constellation of regions associated with live face gaze and chord progressions was found to be synchronous across brains consistent with cooperative sharing of social information (Hasson et al., 2012; Hamilton, 2021). These specific regions include somatosensory association cortices, visual cortex, dorsal lateral prefrontal cortex, supramarginal gyrus, middle and superior temporal gyri, and premotor cortex and have all been implicated in social systems (Carter and Heuttel, 2013). Although our neural coupling findings are exploratory and descriptive, we expect that cooperative processes between brains may have a “yet to be discovered” role in modulating spontaneous interactive social behaviors (Luft et al., 2022; Koul et al., 2023). We note that the neural pairings are distinguished from control computations where the partners are randomly “scrambled” and suggest that the coherence observed is due to some feature of the live interactive experience.

A theoretical framework for the development of evidence-based therapeutic approaches for the potential benefits of music emerges from these findings. Subjective ratings of connectedness were highest during face-to-face gaze while listening to the chord progressions. It has been previously proposed that the somatosensory association cortex encodes subjective feelings accompanying emotional percepts (Iwamura, 2003; Kragel and LaBar, 2016; Koelsch et al., 2021), and it has also been shown to encode pain from social exclusion as well as empathy for social pain (Kross et al., 2011; Novembre et al., 2015). The role of chord progressions in social connection may be facilitated by its role in enhancing synchrony between individuals, an important mediator of social connection (Stupacher et al., 2017). Additionally, chord progressions may drive or modulate reward processing systems that impact social behavior such as the prefrontal cortex and nucleus accumbens (Salimpoor et al., 2011; Ferreri et al., 2019). Prediction error, a key component of reward, may be an important mechanism by which chord progressions serve as a scaffold to drive synchrony between individuals. Previous findings have shown prediction error signals such as the early right anterior negativity (ERAN) are observed when an unsuspected chord is played as part of a chord progression (Loui and Wessel, 2007; Koelsch et al., 2008; Loui et al., 2009; Kim et al., 2011).

Understanding the biological properties that are elicited by music and how these impact the social brain contributes to the development of clinical applications across a variety of medical specialties where music already has demonstrated efficacy (Altenmüller and Schlaug, 2013). Results from the current social music paradigm support the use of tools like music to facilitate social connections. Importantly, the results of this investigation are the first to provide evidence that the right angular gyrus and measures of cross-brain coherence related to live face gaze paired with or without chord progressions and chord progressions paired with or without live faces are associated with social processes and subjective feelings of social connection.

Interpersonal synchrony has previously been studied largely through the lens of joint music making in which both musicians are actively involved in cocreating or engaging in leader-follower dynamics. This research has spanned simple paradigms such as finger tapping to more complex improvisations between multiple musicians (Müller et al., 2013). While hyperscanning using EEG has traditionally been used in these paradigms, fNIRS has recently emerged as a powerful tool for measurement of synchrony or coherence between multiple individuals (Redcay and Leonhard, 2019; Astolfi et al., 2020). In our paradigm, we see enhanced coherence during face-to-face gaze and chord progression conditions compared with the other conditions. While our participants were not actively engaged in making music, we see increased partner-specific coherence during joint music listening. We used a chord progression that has a high prevalence in Western music and creates a mutually shared knowledge framework hypothesized to promote interpersonal synchronization (Hamilton, 2021; Abalde et al., 2024). While participants did not have a shared musical goal, they did have share goal the task of rating connection. This may have also contributed to the frame for increased synchrony. Future research utilizing similar paradigms in which musicians and non-musicians share musical goals in a joint music-making task will further elucidate how active music making may enhance these interpersonal synchrony processes.

The current loneliness epidemic (Murthy, 2021) highlights the critical need for solutions and motivates rigorous investigations aimed at understanding the relationships between features of music and dyadic interactions that might be applied in therapeutic settings (Groarke and Hogan, 2016; Holt-Lunstad, 2021). As group therapy becomes a mainstay in treatment, music that drives synchrony and facilitates group connections could be utilized as an evidence-based intervention to enhance group treatment efficacy. Understanding the features of music necessary to drive neural synchrony and subjective social connection enhance these potential options for music-based interventions (Savage et al., 2021; Chen et al., 2022). Future work will aim to disentangle how specific features of chord progressions such as frequency and rhythm drive neural synchrony and shape social connection.

### Limitations and future directions

Measurements of perceived social connection are inherently subjective, span a range of possible personal interpretations, and can be conceptualized as multidimensional (Verhagen et al., 2025). The typical measurement strategies range from global measurements of connection to partner-specific or interaction-specific measurements (Okabe-Miyamoto et al., 2024). In our experiments, our rating of connection includes both a partner and interaction-specific measurement of connection. This is based on a person's subjective judgment of their social relatedness to their partner during each condition (Plackett et al., 2024). In our experiments, participants rate subjective connectedness on a Likert scale. This measurement of social connection has variable individual assumptions, priorities, and implications (Baek et al., 2025). In addition, factors such as emotional valence or perceived congruence mediate one's subjective sense of connection. For example, it is unclear to what extent participants perceived the various conditions as pleasant or unpleasant. Future studies will employ a multivariate approach to measuring social connectedness using multiple approaches.

### Conclusion

Music is universally appreciated as a promoter of social bonding and a potential therapeutic for conditions of social isolation. Development of an evidence-based theoretical framework for a link between neural systems that underlie social behavior and specific features of music such as consonant chord progressions advance potential applications. Dyadic imaging techniques, a live social interaction paradigm, and music conditions with and without a prevalent chord progression were applied to test the hypothesis that listening to chord progressions promotes social bonding and upregulates social neural circuitry. Subjective ratings of social connectedness, neural measures of cross-brain synchrony, and increased activity in the right angular gyrus, dorsal somatosensory association cortex, and DLPFC (components of the social system) support the hypothesis that predictable musical chord progressions are a salient musical feature that upregulates social neural systems and social behaviors such as gaze at live in-person faces. These findings create an evidence-based framework for future use of musical chord progressions to possibly treat symptoms of social disconnection and isolation.

## Data Availability

The datasets for this study are available from the Yale Dataverse Repository: https://doi.org/10.60600/YU/YD0KXW.
